# Keratinocyte‐Associated Biomarkers Reveal Pathogenic Mechanisms in Acne

**DOI:** 10.1096/fba.2025-00255

**Published:** 2026-02-03

**Authors:** Sini Cai, Yinjing Lin, Heng Xie, Xian Ao, Qiwei Liu, Lining Huang

**Affiliations:** ^1^ Medical Cosmetic Center of Dermatology Hospital of Southern Medical University, Guangdong Provincial Dermatology Hospital Guangzhou China; ^2^ Department of Dermatology The First Affiliated Hospital of Jinan University & Jinan University Institute of Dermatology Guangzhou China; ^3^ The Second Affiliated Hospital of Guangzhou University of Chinese Medicine Guangzhou China

**Keywords:** acne, biomarkers, keratinocyte, machine learning, single‐cell RNA sequencing

## Abstract

The cellular and molecular complexity of acne pathogenesis has hindered progress toward effective targeted therapies. While keratinocytes are known to influence skin inflammation, their precise transcriptional programs and regulatory circuitry in acne remain unclear. We developed an integrative computational framework that combines single‐cell RNA sequencing (scRNA‐seq), gene co‐expression network analysis (WGCNA), and two complementary machine learning algorithms (SVM‐RFE, LASSO) to identify disease‐relevant biomarkers. We mapped acne lesion cellular composition, reconstructed keratinocyte differentiation trajectories, and integrated miRNA–transcription factor–drug interaction networks to link molecular signatures to potential interventions. We uncovered marked keratinocyte heterogeneity and enriched late‐stage pro‐inflammatory states in acne lesions, accompanied by increased macrophage/monocyte and T cell infiltration. Six keratinocyte‐associated biomarkers (PYGL, C10orf99, C12orf75, S100A2, PI3, CARD18) were identified, achieving high diagnostic accuracy (AUC > 0.85). Functional enrichment connected these genes to cytokine and chemokine signaling, while regulatory analysis revealed upstream modulators (hsa‐let‐7b‐5p, FOXC1). Drug–gene network mapping suggested repurposing potential for cyclosporin A and valproic acid. In conclusion, our study delineates a keratinocyte‐centered molecular signature that shapes acne pathogenesis and provides potential therapeutic biomarkers.

## Graphical abstract

The study workflow integrates scRNA‐seq and bulk RNA‐seq data to characterize acne pathogenesis. Step 1 identifies expanded keratinocyte populations using single‐cell analysis. Step 2 filters candidate genes via WGCNA and differential expression integration. Step 3 utilizes ensemble machine learning algorithms (Boruta, LASSO, Logistic Regression) to construct a six‐gene diagnostic nomogram. Step 4 validates these biomarkers through immune infiltration, pathway enrichment, and gene‐drug network analyses, establishing a molecular link between keratinocyte differentiation and inflammatory responses.

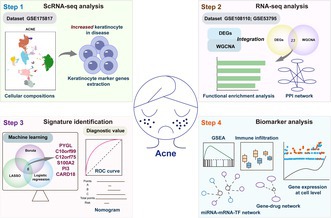

## 1 Introduction

Acne is a persistent inflammatory disease affecting the pilosebaceous unit, with an estimated global prevalence of 9.4%, of which more than 85% of adolescents are impacted [1, 2]. Acne predominantly occurs in areas rich in sebaceous glands, involving the face, back, and chest [3]. Beyond causing physical discomfort, it also exerts a wide‐ranging impact on the mental health of patients, such as anxiety and depression [4]. Although the current acne treatment methods are effective, their options may not be sufficient due to side effects, lack of efficacy, or the financial burden on patients [5]. Therefore, more targeted, effective, and sustainable interventions need to be developed to control the burden of acne. Recent advances emphasize that new insights into the etiology of acne may provide useful information for future therapeutic approaches [6].

The pathophysiology of acne is complex. It is mainly caused by microcomedones resulting from a combination of altered sebum composition, follicular hyperkeratosis, and the growth of *Propionibacterium acnes* ( *P. acnes* ), eventually developing into inflammatory lesion [7]. As the main cell type in the epidermis, keratinocytes play a key role in these pathological processes. On the one hand, androgens stimulate the overexpansion and proliferation of keratinocytes, leading to overproduction of keratin, which exacerbates follicular plugging and hyperkeratosis [8]. On the other hand, follicular blockage triggers the excessive colonization of *P. acnes* in the skin, which can activate the innate immune response through toll‐like receptors 2 and 4 on keratinocytes, further induce the secretion of inflammatory mediators, and thereby promote inflammatory response [9]. Hence, inhibiting the abnormal proliferation of keratinocytes exerts an important therapeutic value in acne. Currently, there is evidence that several drugs such as retinoic acid, trifarotene, and isotretinoin can improve acne by blocking the hyperproliferation of keratinocytes [10, 11, 12]. As a result, an in‐depth understanding of the abnormal behavior of keratinocytes in the pathogenesis of acne is conducive to the development of disease‐targeted therapies [13].

At present, single‐cell RNA sequencing (scRNA‐seq) is widely used in the field of skin [14]. Existing studies have utilized scRNA‐seq to reveal the key signals driving inflammation and hyperkeratosis in acne, providing valuable references for understanding the complex mechanisms of the disease and formulating intervention strategies [15, 16]. However, studies on the use of scRNA‐seq method to explore the heterogeneity of keratinocytes in acne are still lacking. Moreover, the emergence of deep machine learning algorithms has provided insights into the mining of diagnostic biomarkers for skin diseases such as atopic dermatitis and psoriasis [17, 18]. Few articles have reported its application value in acne diagnosis. Therefore, this study combines scRNA‐seq and machine learning methods to establish an acne prediction model based on keratinocyte‐related signature, helping clinicians screen potential risks in a timely manner. This also provides a new perspective for personalized treatment of acne.

## 2 Materials and Methods

### 2.1 Data Downloaded and Processing

All datasets were downloaded from the GEO database. Two acne‐related transcriptome datasets (GSE108110 [19] and GSE53795 [20]) were retrieved. Among them, the GSE108110 dataset contained 18 normal control and 18 disease samples, serving as the training set for WGCNA and key biomarker screening. The GSE53795 included 12 normal control and 12 disease samples, which were used as external data to validate the reliability of biomarkers. Both datasets were generated on the GPL570 platform and used for subsequent analysis after background correction and normalization. Meanwhile, the GSE175817 dataset [21], containing lesional (disease) and non‐lesional (normal) samples from six patients with acne, was utilized for scRNA‐seq analysis. These raw data were preprocessed using the CreateSeuratObject function in the R package Seurat (version 4.3.0) to construct a Seurat object.

### 2.2 Quality Control and Cell Annotation of scRNA‐Seq Data

To ensure the accuracy and reliability of the scRNA‐seq analysis, we first performed quality control of the data using the Seurat software package, excluding genes expressed in less than three cells, cells containing less than 200 genes or more than 10,000 genes, and cells with a percentage of mitochondrial genes greater than 5%. Gene expression of each cell was normalized using the “LogNormalize” method, and then 2000 highly variable feature genes were screened using the “FindVarbleFeatures” function for downstream analysis. We further scaled and centered the feature genes via the ScaleData function, followed by performing dimensionality reduction analysis by the RunPCA function. The top 30 principal components (PCs) were retained for subsequent analysis. “FindNeighbors” and “FindClusters” functions were employed to cluster the dimensionality‐reduced scRNA‐seq data. Cell subgroups were further identified based on known cell marker genes, and the distribution of cell clusters was visualized by the UMAP method. Afterwards, we calculated the difference in the distribution of each cell type in the acne and normal groups.

### 2.3 Differential Expression Analysis of Keratinocyte in Acne and Normal Groups

To extract keratinocyte‐related marker genes in acne, we first analyzed the differences in gene expression between keratinocyte and other cell types. *p* < 0.05 and |avg_log2FC| > 0.25 obtained by Wilcoxon rank‐sum test were used as thresholds to screen keratinocyte‐specific marker genes. Next, differentially expressed marker genes between the acne and normal groups were determined by the Wilcoxon rank‐sum test using the “FindMarkers” function, with *p* < 0.05 and |avg_log2FC| > 0.5 as the screening criteria. These differential genes were utilized as potential markers of acne for subsequent analysis.

### 2.4 Cell Communication

To explore cellular interactions and signaling, we analyzed the cell‐to‐cell communication strength and ligand‐receptor pair by the “Cellchat” package in the disease and normal groups, respectively.

### 2.5 Identification of Keratinocyte‐Related DEGs

Based on the training dataset (GSE108110), the difference of transcriptome data between acne and normal control samples was analyzed by the limma package. |log2FC| > 0.5 and *p* < 0.05 were designated as DEGs and visualized by volcano plot and heat map. Subsequently, these DEGs were integrated with keratinocyte‐related differential marker genes via the Venn method, and the shared genes (named keratinocyte‐related DEGs) were used for subsequent analysis.

### 2.6 WGCNA

WGCNA was applied to screen co‐expressed gene modules and key genes associated with acne in the GSE108110. First, according to the scale‐free topological criterion, the “pickSoftThreshold” function was utilized to determine the optimal soft threshold power β and construct the adjacency matrix. Next, a dynamic tree‐cutting algorithm was used to identify gene modules, and Pearson correlation analysis was employed to associate module signature genes with clinical phenotypes. The modules most relevant to acne were selected. In addition, key genes were screened from these modules using |MM| > 0.8 and |GS| > 0.2 as cut‐off criteria. Here, MM denotes the correlation between the gene and the eigenvalue of the module it belongs to, and GS denotes the correlation between the gene expression level and acne. Finally, the module‐related key genes were integrated with keratinocyte‐related DEGs, and the obtained shared genes were used for further biomarker mining.

### 2.7 Biological Function Analysis and PPI Network Construction

To elucidate the biological significance of the candidate genes, GO‐biological processes (BP) and KEGG enrichment analyses were conducted by clusterProfiler. GO analysis reveals the functional characteristics of the genes. KEGG highlights the regulatory roles of the genes in metabolism, signaling, and disease pathways. A threshold of *p* < 0.05 was used to screen significantly enriched results. Moreover, interactions between proteins encoded by the candidate genes were analyzed using the STRING database, followed by visualization of pairs with confidence score > 0.4 by Cytoscape software.

### 2.8 Screening and Validation of Diagnostic Biomarkers

Three different machine learning algorithms were employed to further identify the optimal diagnostic markers in acne. Boruta is a Random Forest‐based feature selection algorithm, which is analyzed by the “Boruta” package. LASSO implements important feature selection by introducing the L1 regularization method, and this analysis is performed with the “glmnet” package. Univariate Logistic regression utilizes the “glm” function to evaluate the relationship between each feature and the dependent variable, with *p* < 0.05 selected important variables. Variables that co‐existed in the three models were defined as potential biomarkers in acne. Subsequently, in the training (GSE108110) and validation (GSE53795) datasets, differences in the expression levels of these potential markers between disease and normal control samples were estimated. The diagnostic efficacy of these markers was quantified by plotting ROC curves and calculating the area under the curve (AUC). Genes with consistent expression trends and AUC > 0.7 within training and validation sets were selected as acne biomarkers for further model construction.

### 2.9 Establishment of Risk Prediction Model

To visualize the contribution of these biomarkers to acne diagnosis, a nomogram was built using the “rms” package. We also assessed the diagnostic efficacy and clinical value of the nomogram via calibration curve and decision curve analysis (DCA).

### 2.10 Functional Enrichment Analysis of Diagnostic Biomarkers

First, GSEA analysis was performed for each biomarker using the clusterProfiler package to further understand the KEGG pathway enrichment of gene distribution under different phenotypes. Besides, differences in KEGG pathway between acne and normal control samples were assessed by GSVA, and then correlations between biomarkers and differential pathways were calculated. All gene sets were derived from MSigDB. Significance threshold was set at *p* < 0.05.

### 2.11 Immune Infiltration Analysis

Immune response is known to exert a critical role in the pathogenesis of acne. Hence, we analyzed the type and distribution of immune cells in acne and normal control samples. Then, the Wilcoxon rank‐sum test was utilized to compare the differences in cell infiltration between the two groups. Besides, correlations between immune cells as well as between cells and biomarkers were examined, with the help of the Spearman assay.

### 2.12 Creation of miRNA‐mRNA‐TF Network

To comprehensively reveal the regulatory mechanism of biomarkers, the upstream TFs and miRNAs corresponding to the biomarkers were predicted using the miRnet database. Thereafter, the miRNA‐mRNA‐TF network was depicted using Cytoscape software.

### 2.13 Drug Prediction

Diagnostic biomarkers provide reference targets for drug development for diseases. Here, we utilized the DSigDB database to predict biomarker‐related compounds.

### 2.14 Pseudo‐Time Trajectory Analysis of Keratinocyte

To assess the expression status of biomarkers at the single‐cell level, we visualized the expression levels of biomarkers in various cell populations based on the GSE175817 dataset. In addition, R package Monocle 2 was applied to dissect the pseudotemporal trajectories of keratinocyte differentiation. We also focused on the expression patterns of biomarkers on the differentiation trajectory. Furthermore, GO‐BP and KEGG analyses of DEGs in the acne group and the normal group from each differentiation state were conducted using the clusterProfiler package.

## 3 Results

### 3.1 Single‐Cell Transcriptomic Profiling Reveals Expansion of Keratinocytes and Immune Cells in Acne

**FIGURE 1:**
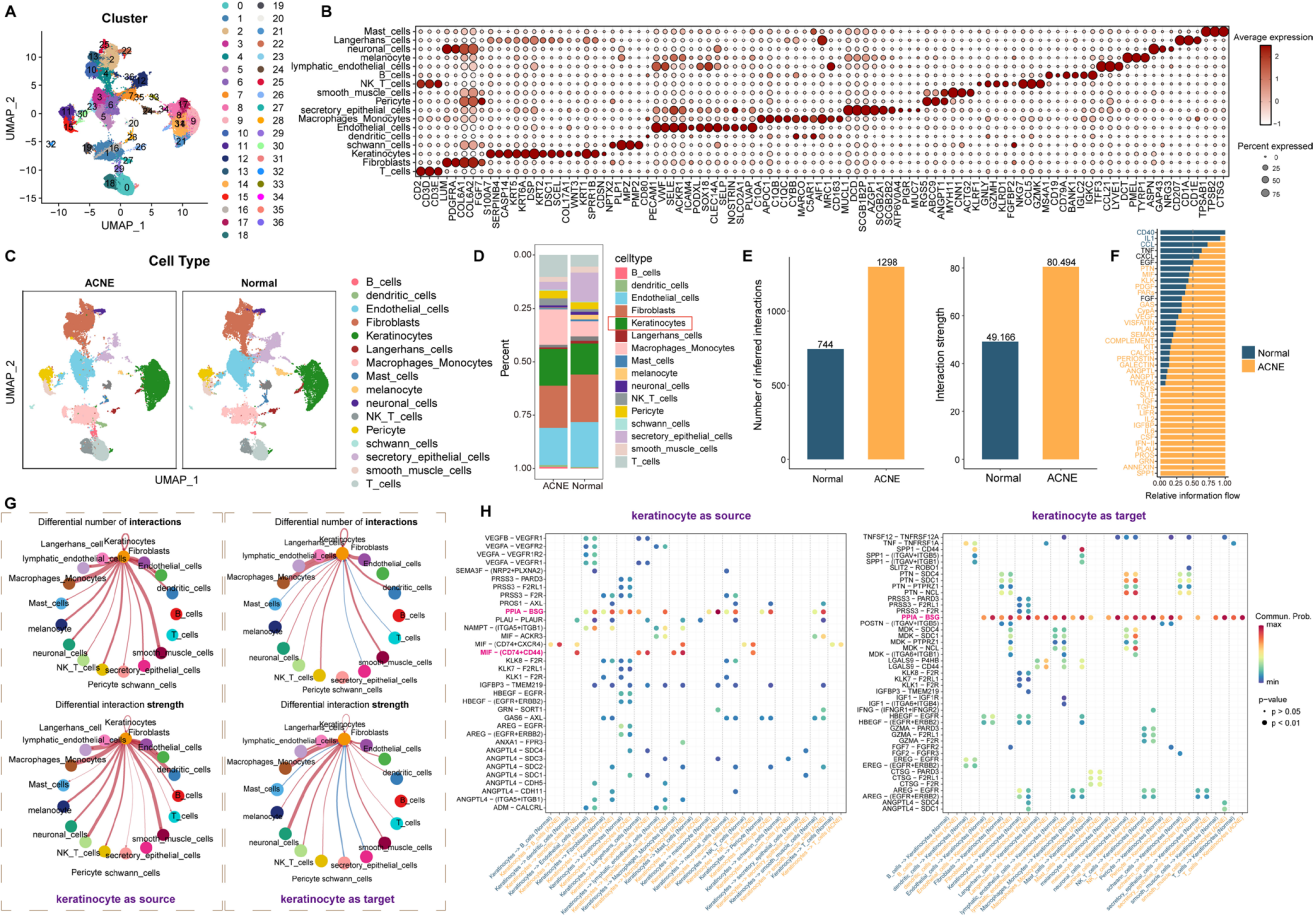
ScRNA‐seq analysis reveals the cellular heterogeneity of acne. (A) UMAP demonstrates that all cells were initially categorized into 37 clusters. (B) Bubble plot illustrating the expression intensity and proportion of canonical marker genes used for cell type annotation. Dot size represents the percentage of cells expressing the gene; color intensity represents the average expression level. (C) UMAP visualization of annotated 16 cell types from acne and normal control samples. (D) Stacked bar chart showing the cellular composition proportions in acne versus normal control samples. (E) Boxplots comparing the number (left) and interaction strength (right) of ligand‐receptor events. (F) Proportion of cellular communication signaling pathways in the normal group and the disease group. (G) Circle plots show the number and strength of communication between keratinocyte and other cell types. (H) Bubble plots reveal ligand‐receptor interactions between different cell types and keratinocyte in normal or acne samples. *p*‐values are indicated by circle size.

To map the cellular landscape of acne lesions, we reanalyzed scRNA‐seq data from six acne and six healthy skin samples (GSE175817). Quality‐controlled data were subjected to UMAP clustering, yielding 37 transcriptionally distinct clusters (Figure 1A). Based on canonical marker expression, these clusters were annotated into 16 skin‐resident cell types, including keratinocytes, fibroblasts, endothelial cells, melanocytes, smooth muscle cells, and multiple immune subsets (Figure 1B).

While both disease and normal control samples contained the same cell categories, their proportions differed significantly. Keratinocytes were expanded in acne, accompanied by higher frequencies of macrophages/monocytes and T cells (Figure 1C,D). Analysis of the cell–cell communication network revealed enhanced connectivity in acne lesions (Figure 1E), with several pro‐inflammatory signaling routes—including IL‐2, IL‐6, IFN‐γ, and TGF‐β—showing elevated activity (Figure 1F).

Focusing on keratinocyte interactions, we observed particularly strong links with endothelial cells, macrophages/monocytes, and smooth muscle cells (Figure 1G). Among the ligand–receptor pairs, PPIA–BSG and MIF–(CD74 + CD44) were notably upregulated in acne, mediating bidirectional signaling between keratinocytes and immune or vascular compartments (Figure 1H).

### 3.2 Keratinocyte‐Focused Analysis Identifies Acne‐Associated Hub Genes and a Six‐Gene Diagnostic Signature

**FIGURE 2:**
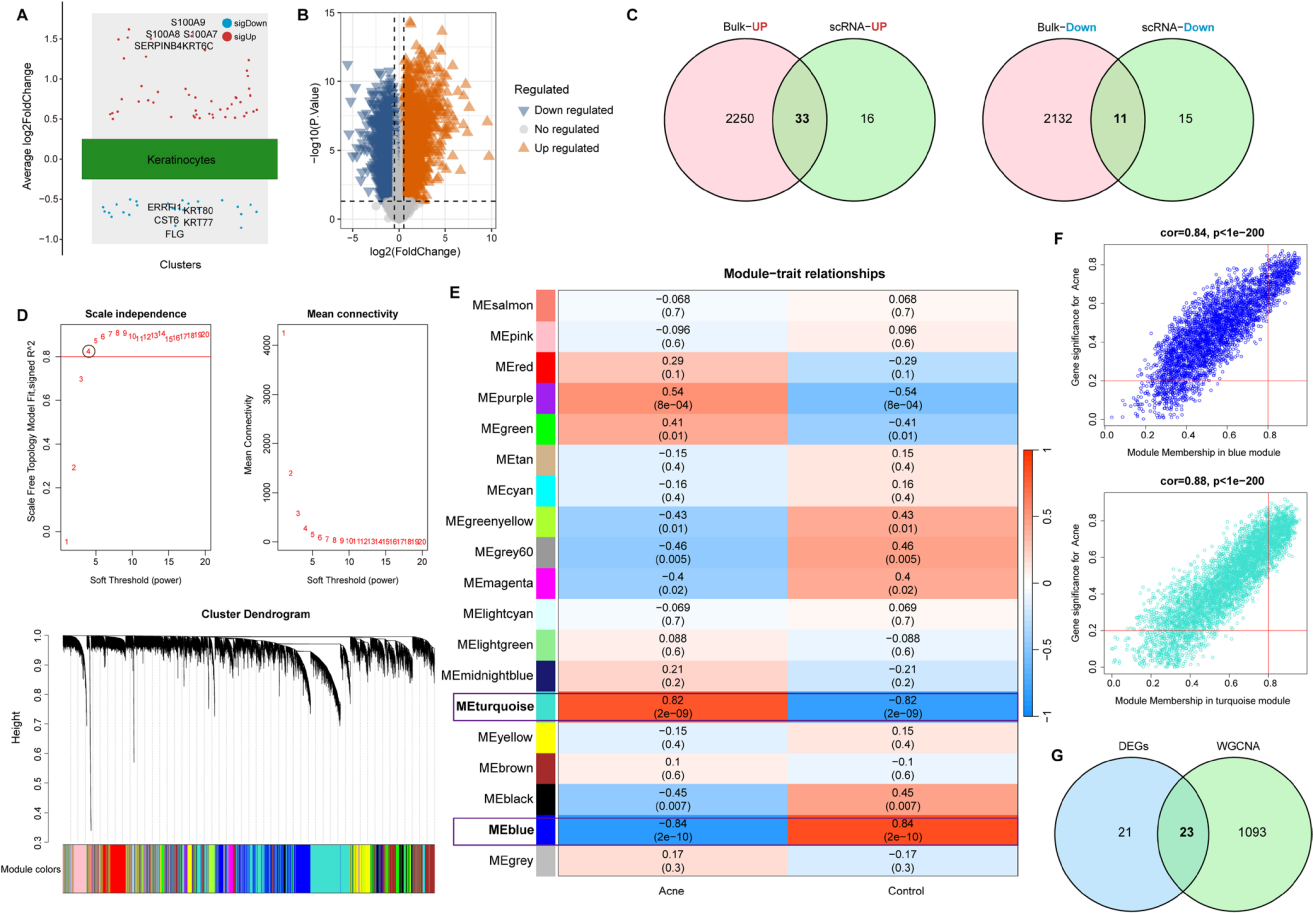
Identification of keratinocyte‐related candidate genes. (A) Volcano plot of DEGs in keratinocytes (acne vs. Normal control). Red points: upregulated genes; blue points: downregulated genes. (B) Volcano plot shows differentially expressed genes between acne and normal control samples. (C) Venn diagram reveals the intersection of genes between keratinocyte‐related markers and DEGs. (D) Soft‐thresholding power *β* = 4 to ensure that the network is scale‐free (above). Cluster dendrogram (below) of the WGCNA. Module colors below the dendrogram indicating gene clustering. (E) Heatmap shows the relationship between module and trait. Turquoise and blue modules are closely related to acne. (F) Scatter plot reveals the relationship between gene significance (GS) and module membership (MM) in the turquoise and blue modules. (G) Venn diagram shows the shared genes between module genes and keratinocyte‐related DEGs.

Given the pronounced keratinocyte expansion, we investigated their transcriptional alterations in detail. A total of 1926 keratinocyte marker genes were identified, of which 75 were differentially expressed between acne and normal control samples (49 upregulated, 26 downregulated; Figure 2A). Independent differential expression analysis of bulk RNA‐seq data (GSE108110) yielded 4426 DEGs, and intersection with keratinocyte markers revealed 44 shared genes (Figure 2B,C).

**FIGURE 3:**
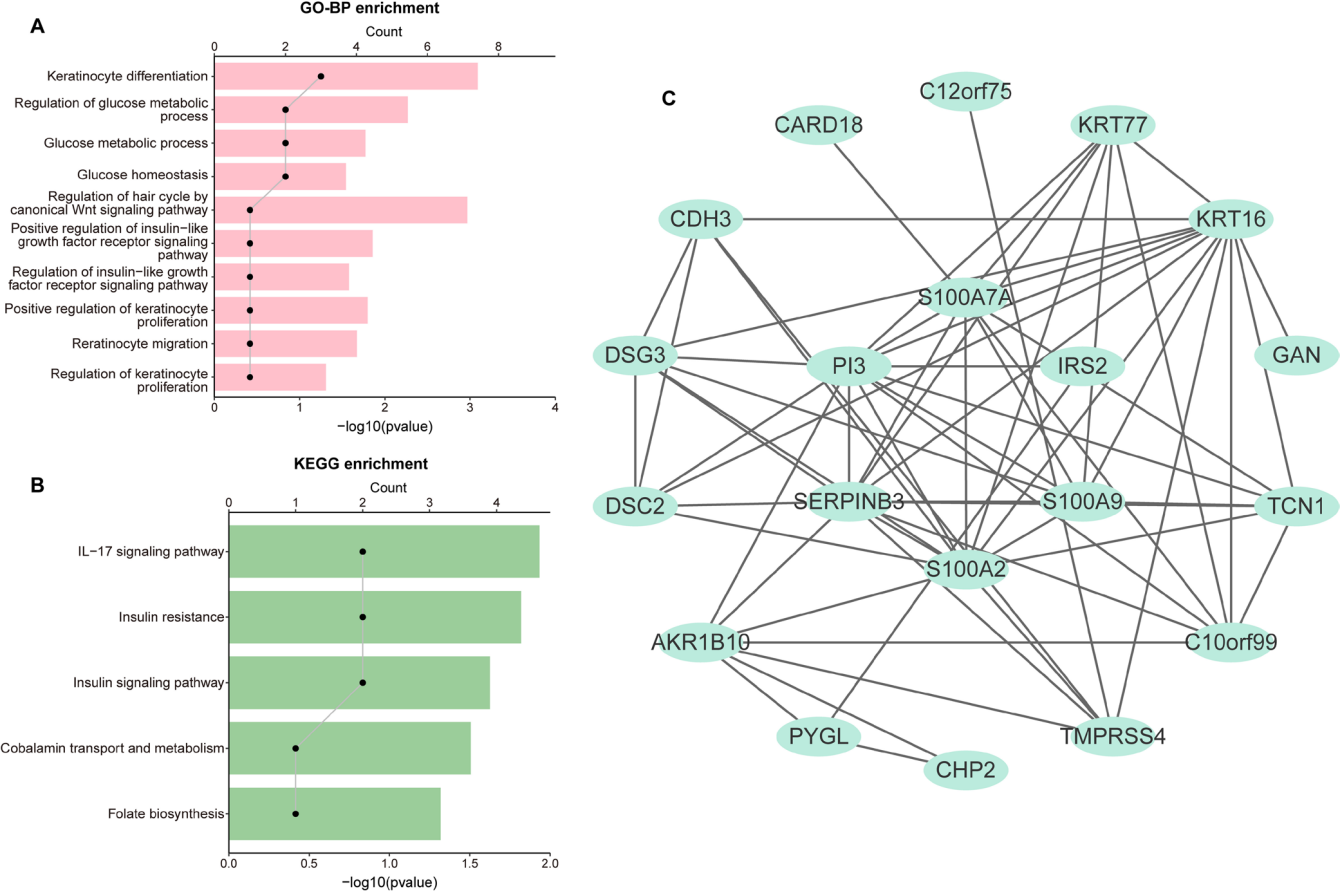
Biological functions and PPI analysis of keratinocyte‐related candidate genes. (A) Bar chart of the top 10 enriched Gene Ontology (GO) Biological Process terms. (B) Bar chart of the top 5 enriched Kyoto Encyclopedia of Genes and Genomes (KEGG) pathways. (C) PPI network constructed using the STRING database and visualized via Cytoscape. Nodes represent proteins; edges represent interactions.

WGCNA identified 18 co‐expression modules, with the turquoise and blue modules showing the strongest correlation with acne (Figure 2D,E). From these, 1116 genes were extracted and intersected with keratinocyte‐associated DEGs, narrowing the list to 23 candidates (Figure 2F,G). GO enrichment linked these genes to keratinocyte differentiation and regulation of proliferation, while KEGG pathways included IL‐17 signaling and insulin signaling (Figure 3A,B).

**FIGURE 4:**
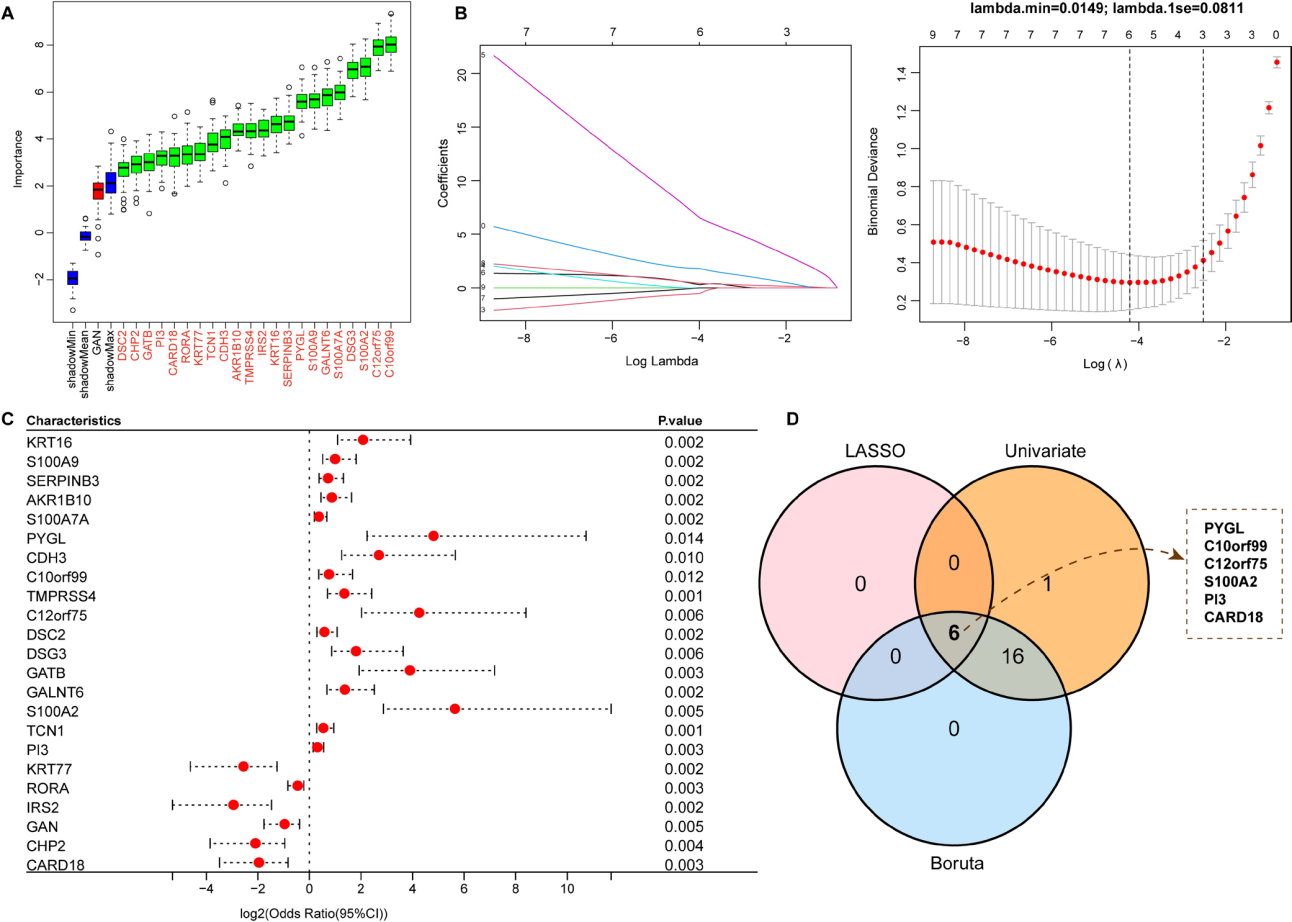
Screening biomarkers for acne by machine learning algorithms. (A) Identification of 22 important variables via the Boruta method. (B) LASSO algorithm identifies 6 significant genes. (C) Forest plot of univariate logistic regression identifying 23 hub genes (Odds Ratio with 95% Confidence Interval). (D) Venn diagram of 6 shared genes among three machine learning algorithms.

**FIGURE 5:**
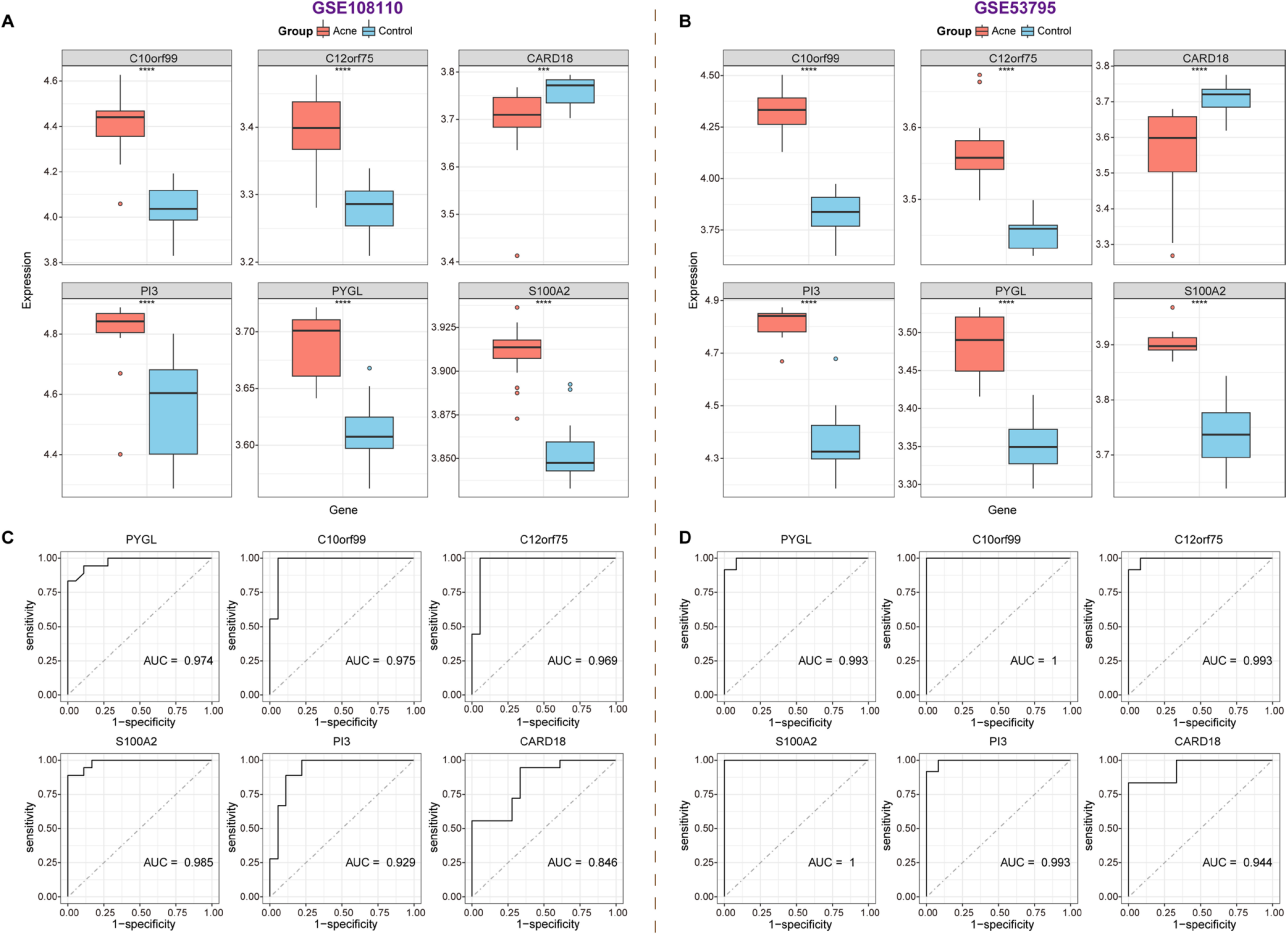
Validation of expression levels and diagnostic values of six biomarkers. (A and B) Expression levels of six genes in acne and normal control samples from training (GSE108110) and validation datasets (GSE53795). ***p < 0.001; ****p < 0.0001. (C and D) ROC curves show the diagnostic accuracy of these biomarkers based on training and validation datasets. AUC, Area Under the Curve.

**FIGURE 6:**
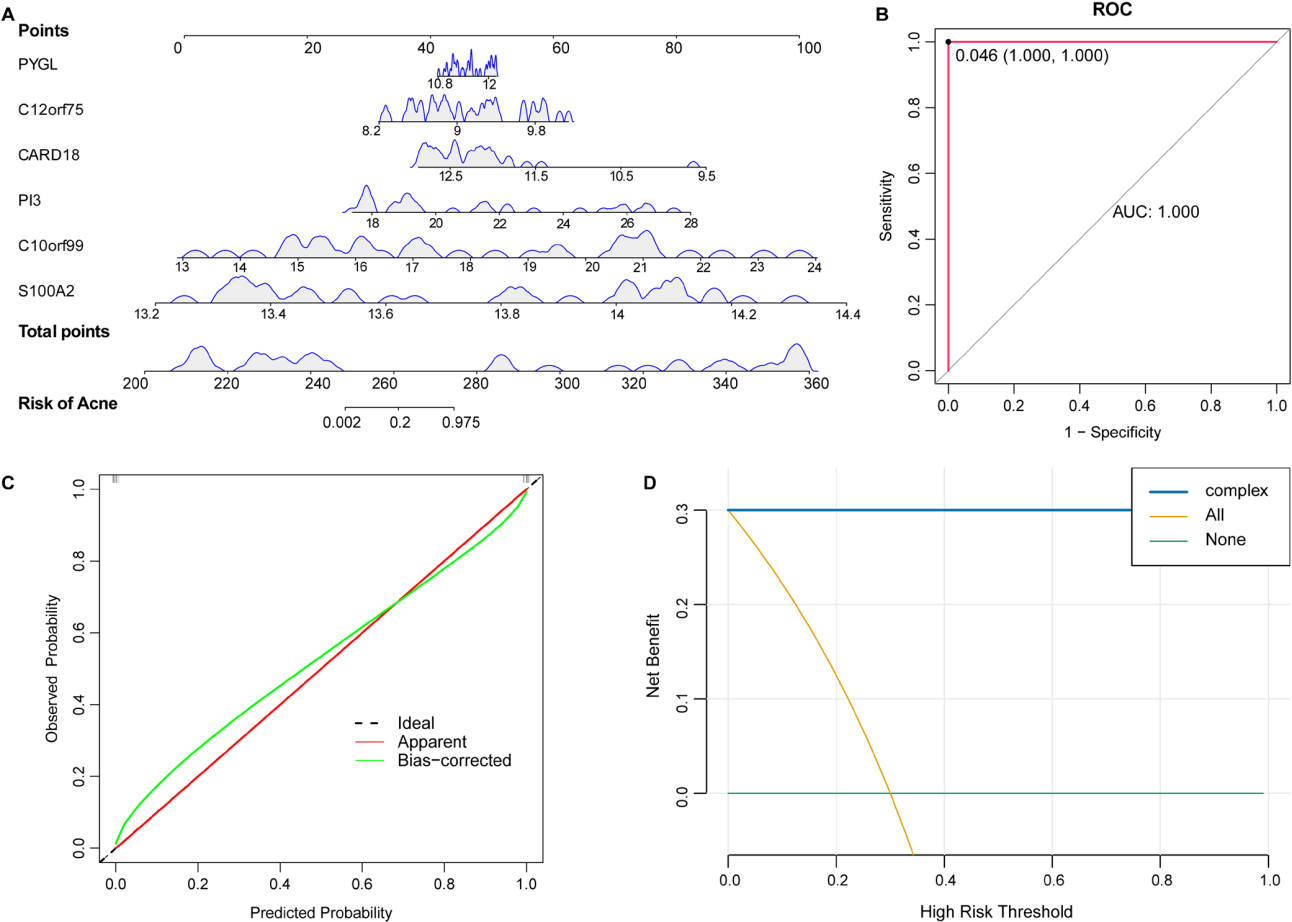
Construction and verification of nomogram model generated by six genes. (A) Nomogram model based on six biomarkers. (B) ROC curve evaluates the predictive performance of the nomogram. (C) Calibration curve assessing the agreement between predicted probabilities and observed outcomes. The 45‐degree dotted line represents perfect prediction. (D) Decision Curve Analysis (DCA) estimating the clinical net benefit of the model across threshold probabilities.

The 23 candidates were subjected to three independent feature selection approaches: Boruta, LASSO regression, and univariate logistic regression (Figure 4A–C). Six genes—PYGL, C10orf99, C12orf75, S100A2, PI3, and CARD18—were consistently selected across methods (Figure 4D). In both training (GSE108110) and validation (GSE53795) datasets, the first five were upregulated and CARD18 downregulated in acne (Figure 5A,B). Each gene achieved an AUC > 0.80 in ROC analysis (Figure 5C,D). A multigene nomogram integrating these markers exhibited perfect discrimination (AUC = 1) and excellent calibration across datasets, with decision curve analysis suggesting high potential clinical utility (Figure 6A–D).

### 3.3 Functional Convergence on Inflammatory Signaling Pathways and Immune Microenvironment Remodeling

**FIGURE 7:**
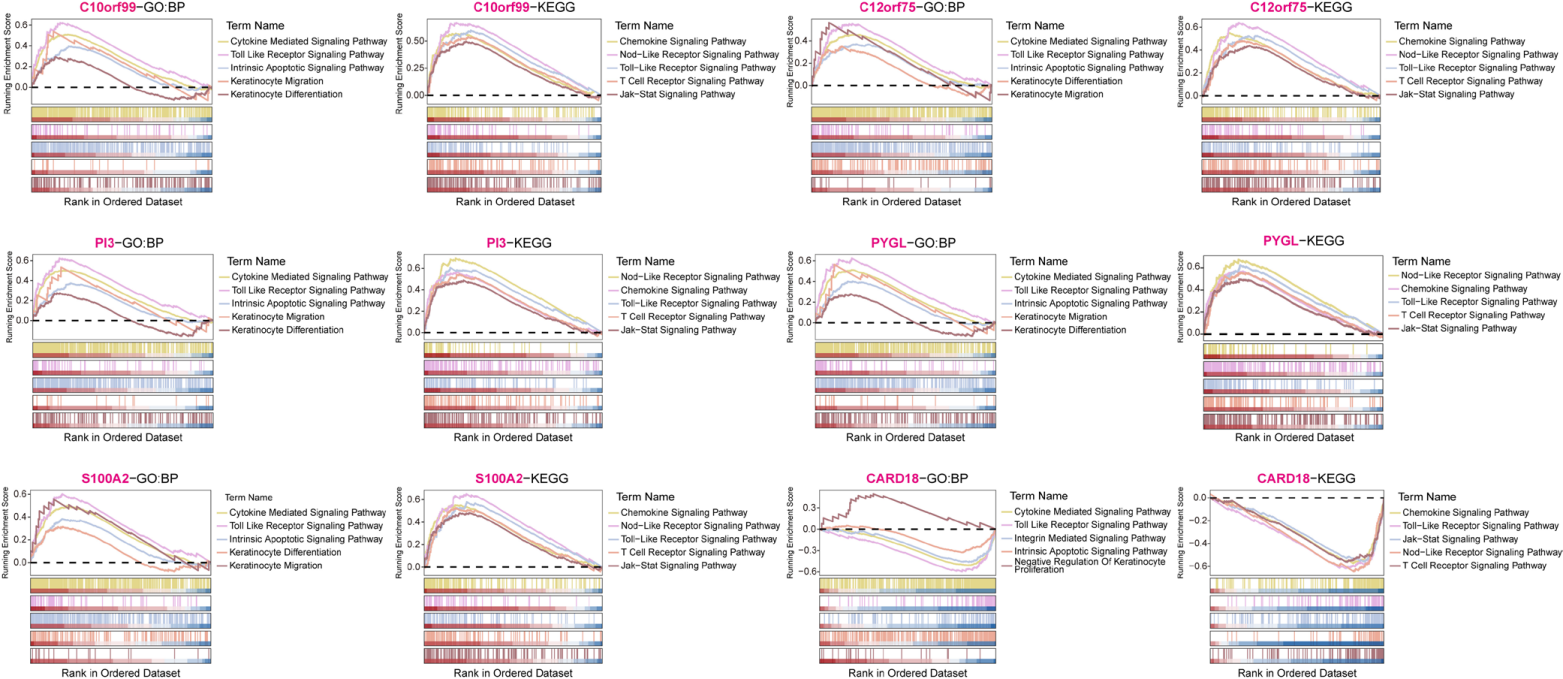
Gene Set Enrichment Analysis (GSEA) of the six biomarkers. Ridgeline plots showing the top enriched GO Biological Process terms and KEGG pathways associated with single‐gene expression levels. Peaks represent the density of fold‐change distribution.

To explore the functional roles of these six genes, we performed GSEA and GSVA. GSEA linked the markers to multiple GO‐BP terms related to cytokine‐mediated signaling, Toll‐like receptor activation, intrinsic apoptotic signaling, and keratinocyte differentiation/migration (Figure 7). KEGG pathways included chemokine signaling, NOD‐like receptor signaling, T‐cell receptor signaling, and JAK/STAT signaling.

**FIGURE 8:**
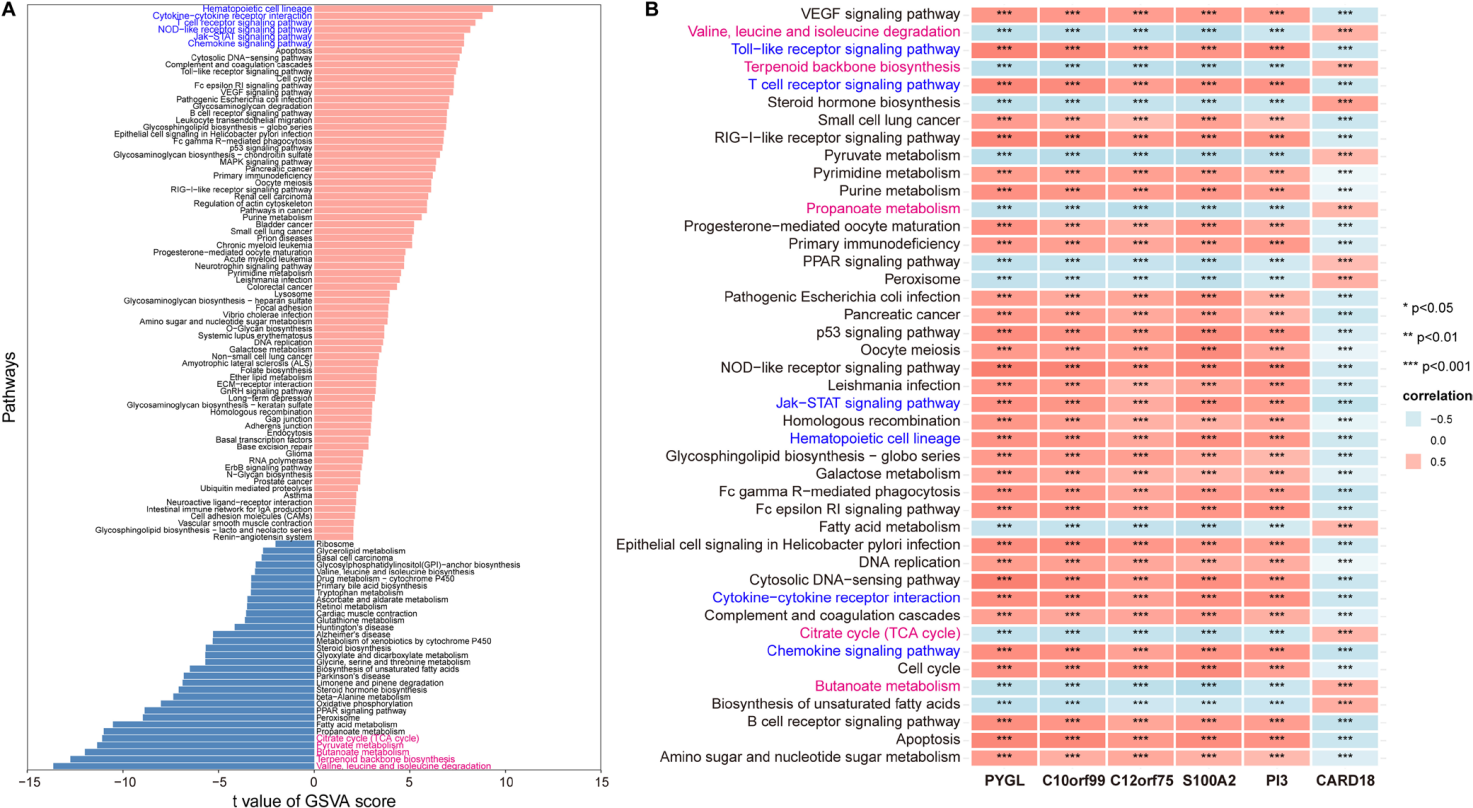
GSVA reveals significantly different KEGG pathways in the acne and normal control samples. (A) Bar plot of differential KEGG pathway activities between acne and normal control samples. Red: upregulated in acne; Blue: downregulated. (B) Heatmap illustrating the correlation between the six biomarkers and pathway activity scores. *p*‐values derived from Pearson correlation analysis (*p < 0.05, **p < 0.01, ***p < 0.001).

GSVA demonstrated that in acne, inflammatory pathways such as hematopoietic cell lineage, cytokine–cytokine receptor interaction, T‐cell receptor signaling, NOD‐like receptor signaling, and chemokine signaling were consistently upregulated, whereas several metabolic pathways—including branched‐chain amino acid degradation, butanoate metabolism, pyruvate metabolism, and the citrate cycle—were attenuated (Figure 8A). Correlation analysis revealed that upregulated biomarkers were positively associated with activated inflammatory pathways and negatively correlated with suppressed metabolic modules (Figure 8B).

**FIGURE 9:**
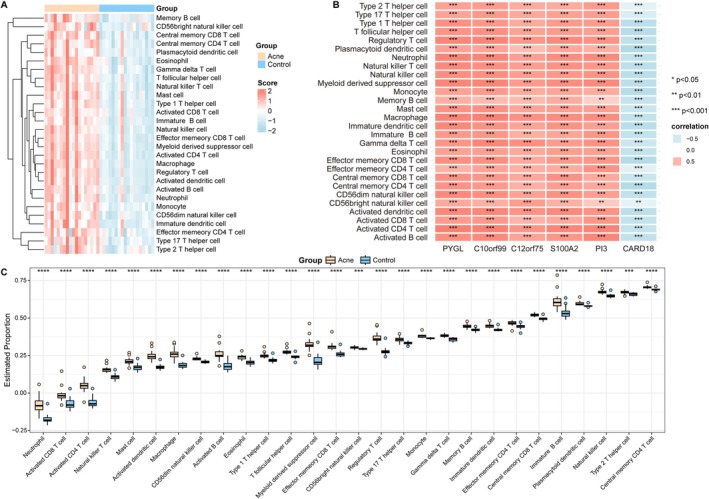
Immune cell infiltration analysis. (A) Heatmap indicates different patterns of immune cell infiltration between acne and normal control groups. (B) Heatmap shows correlation between six genes' expression and immune cell infiltration. (C) Boxplot indicates the difference of immune infiltration between acne and normal control samples. **p < 0.01; ***p < 0.001; ****p < 0.0001.

Immune cell deconvolution confirmed broad increases in infiltrating immune populations in acne (Figure 9A). High expression of PYGL, C10orf99, C12orf75, S100A2, and PI3 strongly correlated with macrophage, neutrophil, and T‐cell abundance, whereas CARD18 expression correlated inversely (Figure 9B,C). These data suggest that the six‐gene signature marks a keratinocyte state that favors immune recruitment and maintenance of an inflammatory milieu.

### 3.4 Regulatory Mechanisms of Biomarkers

**FIGURE 10:**
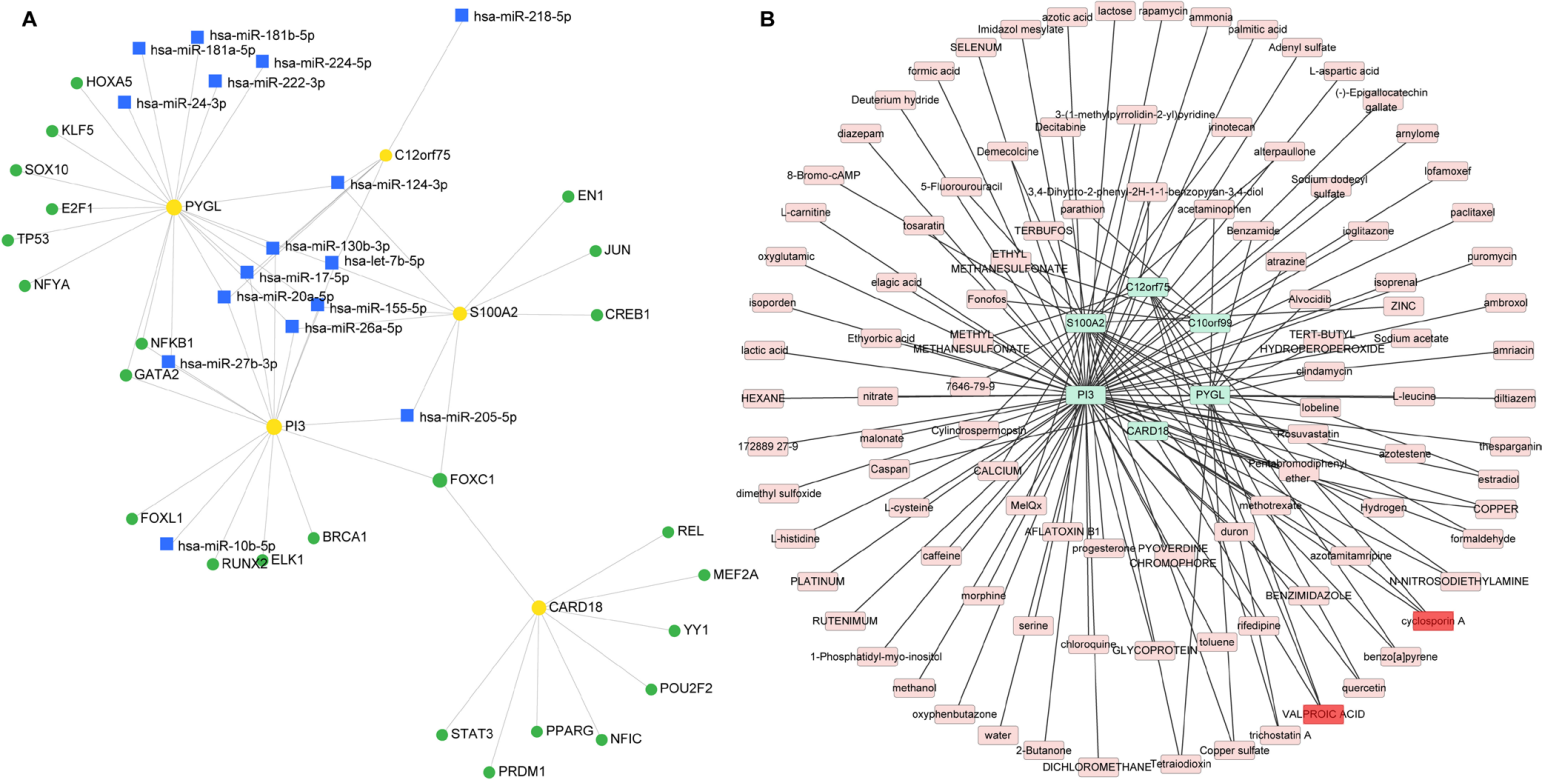
Regulatory mechanisms and drug prediction. (A) miRNA‐mRNA‐TF network. Yellow dots, blue squares, and green dots represent mRNAs, miRNAs, and TFs, respectively. Edges indicate experimentally validated interactions. (B) Gene‐drug network. Pink nodes: predicted drug candidates; Green nodes: mRNA targets.

Using the miRNet database, we constructed a multi‐layered regulatory network to explore upstream controllers of the identified biomarkers. The resulting miRNA‐mRNA‐TF network (Figure 10A) comprises 5 key biomarkers (PYGL, C12orf75, S100A2, PI3, CARD18), 16 miRNAs, and 24 transcription factors interconnected by 61 edges. In the drug‐gene interaction network (Figure 10B), 104 compounds were predicted to target the identified hub genes. To prioritize candidates with multi‐target potential, we screened for drugs with the highest connectivity (degree ≥ 3). Cyclosporin A and Valproic Acid emerged as the top candidates, each capable of modulating three distinct biomarkers.

### 3.5 Single‐Cell Validation and Keratinocyte Trajectory Analysis Pinpoint Late‐Stage Differentiation Bias in Acne

**FIGURE 11:**
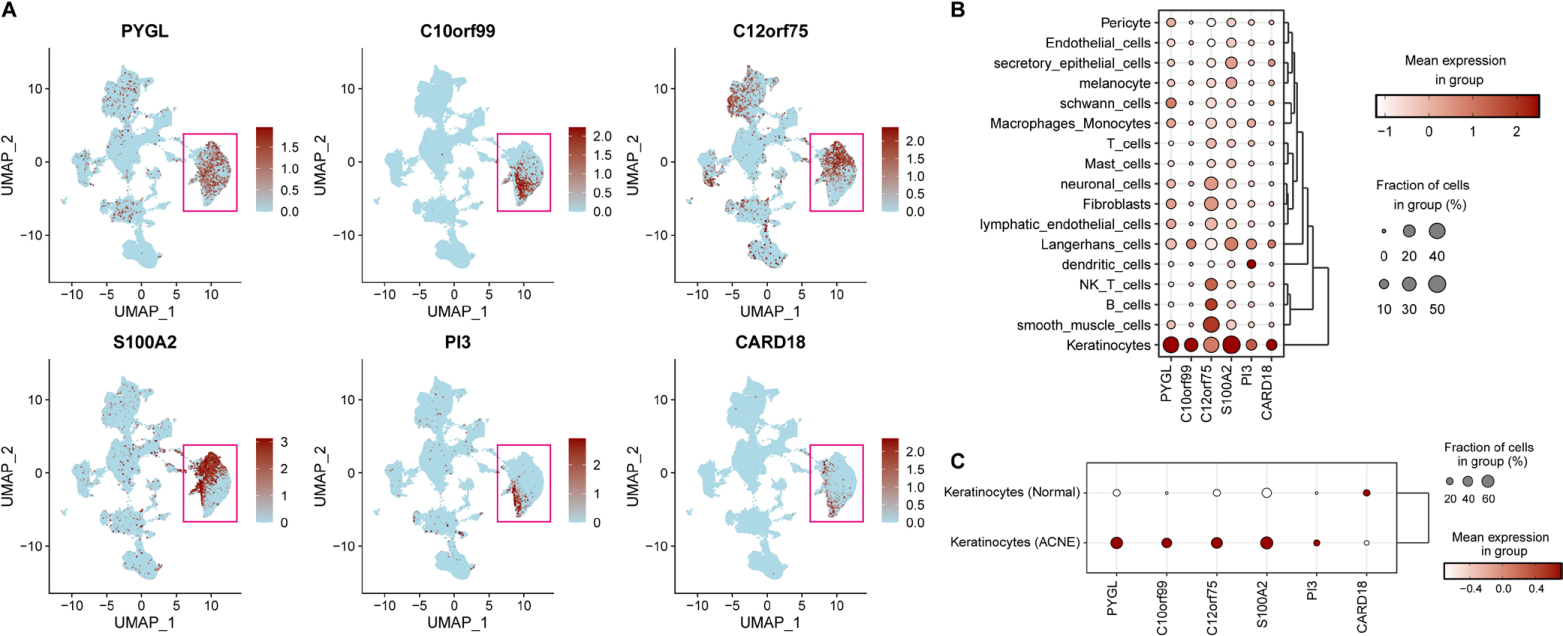
Validation of expression levels of six biomarkers at scRNA‐seq level. (A) Expression of six genes in various cell types by scRNA‐seq analysis. (B) Bubble plot shows the expression levels of genes in different cell types. (C) Bubble plot shows the expression levels of six genes in keratinocytes from disease and normal control samples.

Single‐cell validation confirmed that the six signature genes were predominantly expressed in keratinocytes, with higher expression of PYGL, C10orf99, C12orf75, S100A2, and PI3, and lower CARD18 expression in acne lesions (Figure 11A–C).

**FIGURE 12:**
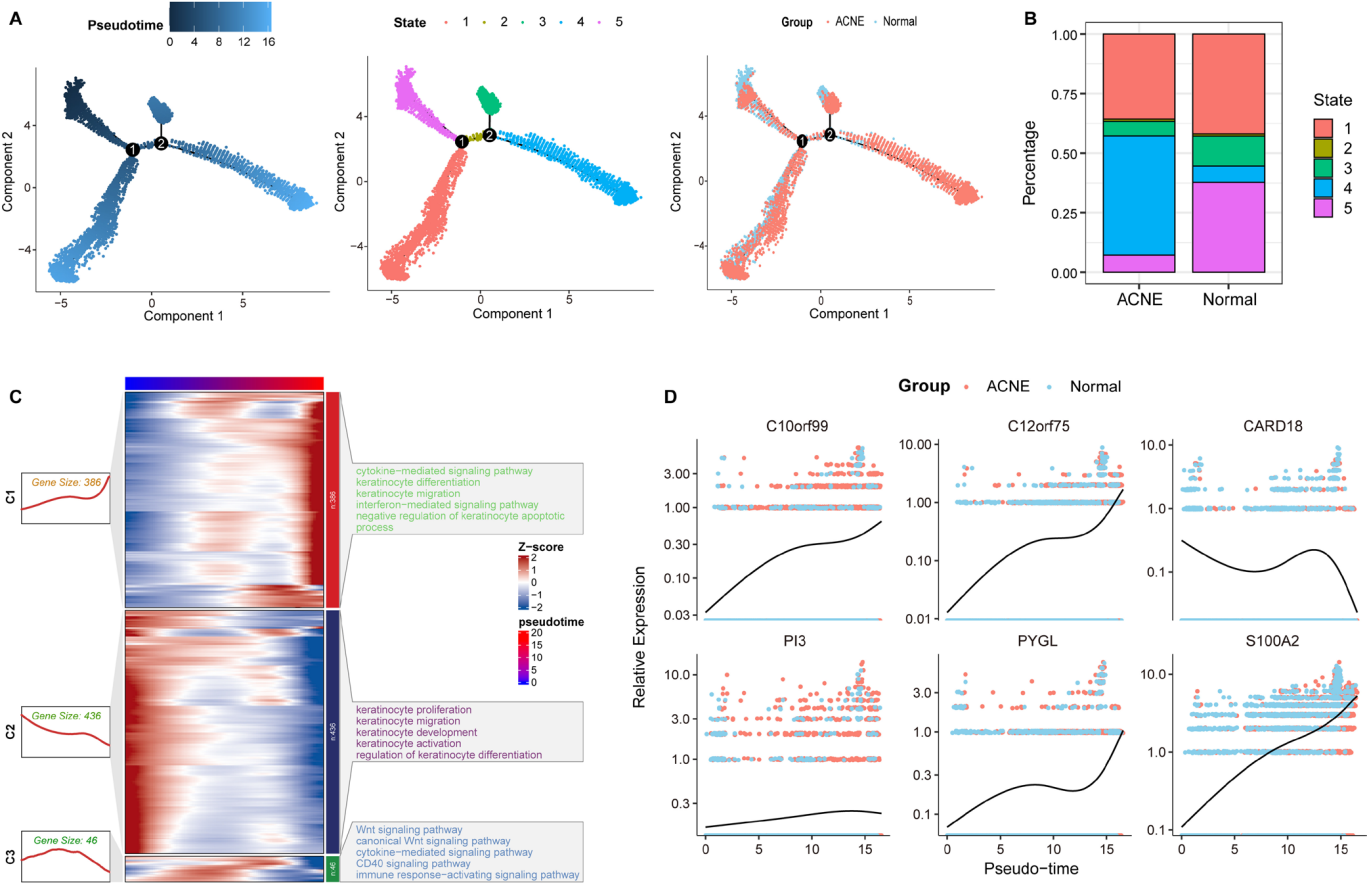
Analysis of the differentiation status of keratinocyte in acne and normal control samples. (A) Pseudo‐time analysis of keratinocytes from acne and normal control samples. (B) Distribution ratios of different keratinocyte differentiation states in acne and normal control samples. (C) Heatmap shows the dynamic changes in gene expression and KEGG pathways along the pseudo‐time. (D) Dynamic expression of six genes along the pseudo‐time in normal control (blue) and acne (red) samples.

Pseudotime trajectory reconstruction identified five keratinocyte differentiation states (Figure 12A). In healthy skin, most keratinocytes occupied early‐stage states, whereas in acne lesions they were skewed toward late differentiation stages (Figure 12B). Late‐stage keratinocytes exhibited enrichment for cytokine‐mediated signaling, keratinocyte differentiation, and migration pathways (Figure 12C). Along the pseudotime axis, PYGL, C10orf99, C12orf75, PI3, and S100A2 increased progressively, while CARD18 decreased—mirroring their expression in bulk datasets (Figure 12D). These results indicate that acne is associated with a shift toward a pro‐inflammatory late keratinocyte differentiation program, marked by the six‐gene signature.

## 4 Discussion

This study provides a single‐cell–resolved map of keratinocyte‐associated molecular alterations in acne and defines a six‐gene diagnostic panel with discriminatory power. By linking keratinocyte expansion and inflammatory rewiring to specific biomarkers and signaling pathways, we offer both mechanistic insights and potential tools for early detection. These findings establish a framework for understanding how keratinocyte differentiation and pathogenic inflammation are molecularly coupled in acne lesions.

While the individual contributions of activated keratinocytes [22, 23] and infiltrating macrophages [24] or T cells [19] to acne are well‐documented, the molecular mechanisms coupling these compartments remain incompletely defined. Our analysis suggests that these cell types are functionally linked via the PPIA‐BSG signaling axis, rather than simply co‐existing in the inflammatory niche. In contrast to classical models that emphasize Cutibacterium acnes as the primary trigger for immune recruitment [25, 26], the presence of PPIA‐BSG interactions—previously characterized in tumor microenvironments [27, 28]‐indicates an intrinsic pathway capable of sustaining cutaneous inflammation. This finding refines the current understanding of keratinocytes, framing them not merely as a physical barrier affected by follicular occlusion, but as active immune modulators driving the persistence of acne lesions through direct signaling to CD4^+^ T cells and macrophages.

Through a keratinocyte‐focused integrative pipeline combining differential expression analysis, WGCNA, and three machine‐learning algorithms, we identified six high‐confidence biomarkers: PYGL, C10orf99, C12orf75, S100A2, PI3, and CARD18. Although direct evidence linking these genes to acne is limited, literature in related inflammatory dermatoses informs potential mechanisms. Beyond its established role in psoriatic keratinocyte inflammation [29], PYGL upregulation here suggests that cell‐autonomous metabolic reprogramming is a primary driver of acne initiation, predating bacterial involvement. This enhanced glycogenolytic flux is particularly significant given the clinical dependence of acne on the GH/IGF‐1 axis—evidenced by the virtual absence of lesions in IGF‐1‐deficient Laron syndrome [30] versus their severity in acromegaly [31]. We propose that PYGL‐driven glucose availability provides the requisite substrate to support this IGF‐1 phenotype. High glucose flux likely creates a state of “metabolic permissiveness” [32] that potentiates the PI3K/Akt/SREBP‐1 axis described by Smith et al. [33], thereby amplifying the twin pathologies of sebocyte lipogenesis and ductal hyperproliferation. Concurrently, this hypermetabolic state may independently prime the NLRP3 inflammasome via mitochondrial ROS. Consequently, we hypothesize that localized metabolic dysregulation establishes a nutrient‐rich, inflamed niche that invites subsequent C. acnes colonization, highlighting upstream metabolic shifts as a viable therapeutic target.

C10orf99 modulates epidermal barrier formation and induces inflammatory cascades [34]. C12orf75 may influence immune responses by modulating NK cell activity [35]. S100A2 regulates keratinocyte differentiation and correlates with severity in inflammatory skin disease [36]. PI3, normally absent from healthy epidermis, is induced in inflamed keratinocytes [37]. CARD18 attenuates IL‐1β maturation, acting as a brake on inflammatory signaling [38, 39].

The clinical relevance of these markers is supported by their strong diagnostic performance, both individually and in a multigene nomogram. Pathway analyses converge on chemokine‐ and cytokine‐mediated signaling, NOD‐like receptor activity, and other canonical inflammatory routes [40, 41, 42], reinforcing their role in amplifying leukocyte recruitment and sustaining cutaneous inflammation. Such molecular specificity positions this six‐gene panel as a promising diagnostic and potentially therapeutic target set for acne.

Trajectory analysis extended these findings by revealing a shift in keratinocyte populations toward late differentiation states in acne lesions. These stages were enriched for cytokine signaling, migratory programs, and other pro‐inflammatory functions, with PYGL, C10orf99, C12orf75, S100A2, and PI3 following a progressive upregulation along pseudotime, while CARD18 declined. This pattern suggests a possible causal link: dysregulated differentiation may lock keratinocytes into an inflammation‐prone state, perpetuating lesion development.

Regulatory network analyses highlight hsa‐let‐7b‐5p and the transcription factor FOXC1 as upstream modulators. The let‐7b family has been implicated in skin wound repair [43], and FOXC1 is essential for normal keratinocyte maturation, with its downregulation associated with inflammatory dermatitis [44]. Topological analysis ranked Cyclosporin A and Valproic Acid as the top candidates based on their high connectivity. Cyclosporin A has been reported to treat severe, neutrophil‐driven acne fulminans [45], while Valproic Acid was recently shown to modulate the cutaneous immune microenvironment by downregulating pro‐inflammatory cytokines, including IL‐1β and TNF‐α [46]. While these findings remain predictive, they suggest defined molecular entry points for therapeutic intervention.

Several caveats must be acknowledged. First, our conclusions derive from publicly available transcriptomic datasets; functional validation in in vitro and in vivo acne models is essential to confirm causality. Second, keratinocytes were the primary focus of single‐cell trajectory analysis; extending similar lineage reconstructions to immune and stromal elements could yield a more complete view of lesion evolution. Third, the mechanistic links between predicted regulators or candidate drugs and biomarker expression require experimental dissection. Addressing these points will refine our understanding of disease networks and inform translational strategies.

## Author Contributions

S.C. designed the project, analyzed the data and finished the manuscript. Y.L., H.X. and X.A. Provide assistance in data curation and methodology. Q.L. and L.H. designed the project and revised the manuscript. All authors checked and approved the final manuscript.

## Funding

This work was supported by the Miao Pu Scientific Research Fund of the Dermatology Hospital of Southern Medical University (230350ZC).

## Ethics Statement

The authors have nothing to report.

## Conflicts of Interest

The authors declare no conflicts of interest.

## Data Availability

The authors have nothing to report.
